# CD8 coreceptor-mediated focusing can reorder the agonist hierarchy of peptide ligands recognized via the T cell receptor

**DOI:** 10.1073/pnas.2019639118

**Published:** 2021-07-16

**Authors:** Mathew Clement, Lea Knezevic, Tamsin Dockree, James E. McLaren, Kristin Ladell, Kelly L. Miners, Sian Llewellyn-Lacey, Anzelika Rubina, Ore Francis, David K. Cole, Andrew K. Sewell, John S. Bridgeman, David A. Price, Hugo A. van den Berg, Linda Wooldridge

**Affiliations:** ^a^Division of Infection and Immunity, Cardiff University School of Medicine, University Hospital of Wales, Cardiff CF14 4XN, United Kingdom;; ^b^Faculty of Health Sciences, University of Bristol, Bristol BS8 1TD, United Kingdom;; ^c^Immunocore Ltd., Abingdon OX14 4RY, United Kingdom;; ^d^Systems Immunity Research Institute, Cardiff University School of Medicine, University Hospital of Wales, Cardiff CF14 4XN, United Kingdom;; ^e^Immetacyte Ltd., Manchester M13 9XX, United Kingdom;; ^f^Warwick Mathematics Institute, University of Warwick, Coventry CV4 7AL, United Kingdom

**Keywords:** CD8 coreceptor, pMHCI, T cell activation

## Abstract

CD8^+^ T cells are inherently cross-reactive and recognize numerous peptide antigens in the context of a given major histocompatibility complex class I (MHCI) molecule via the clonotypically expressed T cell receptor (TCR). The lineally expressed coreceptor CD8 interacts coordinately with MHCI at a distinct and largely invariant site to slow the TCR/peptide-MHCI (pMHCI) dissociation rate and enhance antigen sensitivity. However, this biological effect is not necessarily uniform, and theoretical models suggest that antigen sensitivity can be modulated in a differential manner by CD8. We used two intrinsically controlled systems to determine how the relationship between the TCR/pMHCI interaction and the pMHCI/CD8 interaction affects the functional sensitivity of antigen recognition. Our data show that modulation of the pMHCI/CD8 interaction can reorder the agonist hierarchy of peptide ligands across a spectrum of affinities for the TCR.

## Significance

Sufficient immune coverage of the peptide universe within a finite host requires highly degenerate T cell receptors (TCRs). However, this inherent need for antigen cross-recognition is associated with a high risk of autoimmunity, which can only be mitigated by a process of adaptable specificity. We describe a mechanism that resolves this conundrum by allowing individual clonotypes to focus on specific peptide ligands without alterations to the structure of the TCR.

CD8^+^ T cells are critical for protective immunity against intracellular pathogens and various tumors. At the molecular level, activation is triggered by foreign or mutated peptide fragments presented on the cell surface by major histocompatibility complex class I (MHCI) molecules, which act as ligands for the somatically rearranged T cell receptor (TCR) and the germline-encoded coreceptor CD8 (1, 2). The clonotypically expressed TCR confers antigen specificity by interacting with the peptide-binding platform of MHCI, which comprises the α1 and α2 domains, whereas the lineally expressed coreceptor CD8 is known to enhance antigen sensitivity by interacting primarily with the α3 domain of MHCI (3–7). This latter interaction is biophysically and spatially independent of peptide-MHCI (pMHCI) engagement via the TCR (8). However, the largely invariant nature of the pMHCI/CD8 interaction does not necessarily translate into a uniform gain of function, and theoretical studies have suggested that antigen sensitivity can be modulated in a differential manner, potentially altering the agonist hierarchy of peptide ligands for any given TCR (9–11).

The pMHCI/CD8 interaction slows the dissociation rate of the TCR/pMHCI interaction (9, 12). Functional sensitivity depends nonmonotonically on this dissociation rate (13), as long as the system is limited by MHCI (10, 14, 15). The nature of this relationship implies that functional sensitivity reaches a maximum at a particular dissociation rate. Strong agonists are relatively insensitive to modulation of the dissociation rate, because the curve has a negligible slope in the vicinity of the optimal value. In contrast, weak agonists are typically characterized by faster dissociation rates, modulation of which markedly alters functional sensitivity (16). Accordingly, the pMHCI/CD8 interaction generally acts to increase agonist potency, maximizing the number of peptide ligands that can be recognized via a given TCR. However, theoretical models predict that ligands with dissociation rates below or close to the optimal value will respond differently, amounting to a differential focusing effect, whereby strong agonists can become less potent at dissociation rates beyond the optimal value. If operative in vivo, such an effect could allow individual clonotypes to focus on salient ligands (9), reconciling the inherent need for cross-reactivity with the inherent need for specificity (17).

We used two monoclonal systems incorporating biophysically defined peptide ligands and variants of MHCI with altered coreceptor-binding properties to test the differential focusing hypothesis experimentally. In line with earlier predictions, we found that modulation of the pMHCI/CD8 interaction reordered the agonist hierarchy of peptide ligands recognized via the TCR.

## Results

To test the notion that coreceptor-mediated effects can alter ligand specificity, we determined the extent to which CD8 modulated functional responses initiated via the MEL5 TCR, which recognizes the heteroclitic Melan-A epitope ELAGIGILTV_26–35/A27L_ (ELA) restricted by HLA-A*0201 (abbreviated from hereon as HLA-A2). Ligand recognition in this system has been characterized previously using surface plasmon resonance (18, 19). Biophysically defined peptide ligands, including a weak agonist (3T), the wild-type epitope (ELA), and a superagonist (FAT), were selected for the purposes of this work to introduce a range of TCR/pMHCI affinities (Table 1). C1R cells expressing HLA-A2 D227K/T228A, which abrogates the coreceptor interaction (20), wild-type HLA-A2, HLA-A2 A245V/K^b^, which enhances the coreceptor interaction (21), or HLA-A2 K^b^, which superenhances the coreceptor interaction (22), were used in parallel to introduce a range of pMHCI/CD8 affinities (Table 2). Importantly, surface plasmon resonance experiments have shown that none of these mutations, namely D227K/T228A, A245V/K^b^, and K^b^, affect the TCR/pMHCI interaction (12, 21).

**Table 1.** TCR/pMHCI dissociation constants and kinetics for agonists of the MEL5 TCR

MHCI	Epitope	k_on_ (M^−1^s^−1^)	k_off_ (s^−1^)	K_D_ (μM)
HLA-A2	EL**T**GIGILTV (3T)	∼1.2 × 10^4^	∼1	82 ± 4
HLA-A2	ELAGIGILTV (ELA)	∼4.4 × 10^4^	∼0.75	17 ± 1
HLA-A2	**FAT**GIGI**I**TV (FAT)	∼1.2 × 10^5^	0.36	3 ± 1
HLA-A2	**I**LAGIGILTV (1I)	∼1.3 × 10^5^	∼1.3	10.2 ± 0.7*

* Surface plasmon resonance data are shown in *SI Appendix*, Fig. S6*A*.

**Table 2.** pMHCI/CD8 dissociation constants for variants of HLA-A2

MHCI	Mutation	K_D_ (μM)
HLA-A2 D227K/T228A	MHCI α3 domain	>10,000
HLA-A2 WT	N/A	137 ± 9.7
HLA-A2 A245V/K^b^	MHCI α3 domain	27 ± 1
HLA-A2 K^b^	MHCI α3 domain	11

N/A, not applicable.

**Fig. 1.**
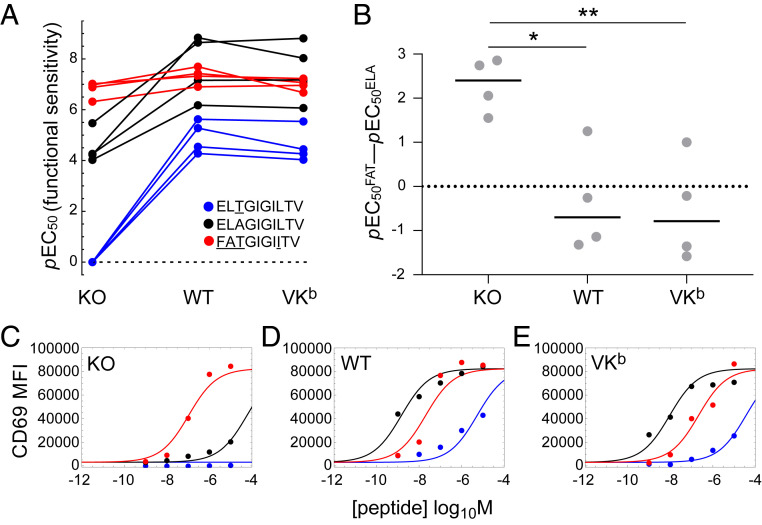
CD8 reorders the agonist hierarchy of peptide ligands that induce the expression of CD69. MEL5 TCR^+^ CD8^+^ J.RT3-T3.5 cells were activated for 6 h with C1R cells expressing comparable levels of HLA-A2 D227K/T228A (KO), wild-type HLA-A2 (WT), or HLA-A2 A245V/K^b^ (VK^b^) pulsed with various concentrations of 3T (blue), ELA (black), or FAT (red). Surface expression of CD69 was measured via flow cytometry. (*A*) Functional sensitivity (*p*EC_50_) for each peptide ligand in the context of each MHCI. Four replicate experiments are shown. The value for 3T in the context of HLA-A2 D227K/T228A was set to zero for graphical purposes and treated as missing data for statistical purposes. *P* < 0.0001 for the ligand effect and *P* < 0.0001 for the MHCI effect (two-way ANOVA with Tukey’s post hoc test). (*B*) The agonist potency of FAT relative to the agonist potency of ELA expressed as *p*EC_50_^FAT^ − *p*EC_50_^ELA^, which is equivalent to the logarithm of the fold difference in functional sensitivity. Four replicate experiments are shown. Horizontal bars indicate median values. *P < 0.05 and **P < 0.01 (one-way ANOVA with Tukey’s post hoc test). (*C*–*E*) Representative peptide titration experiment used to calculate the parameters in *A* and *B*. Curves were fitted in Mathematica. All four replicate experiments are shown in *SI Appendix*, Figs. S1 and S2.

In preliminary experiments, we quantified CD69 on the surface of MEL5 TCR^+^ CD8^+^ J.RT3-T3.5 cells as a measure of activation in response to 3T, ELA, or FAT presented in the context of HLA-A2 D227K/T228A, wild-type HLA-A2, or HLA-A2 A245V/K^b^ (*SI Appendix*, Figs. S1 and S2). Functional sensitivity was determined as the *p*EC_50_ value for each parameter combination (Fig. 1*A*). In the absence of a pMHCI/CD8 interaction (HLA-A2 D227K/T228A), activation was a simple function of TCR/pMHCI affinity (Fig. 1*A* and *SI Appendix*, Figs. S1 and S2). The agonist potencies of 3T and ELA were enhanced in the context of HLA-A2 and HLA-A2 A245V/K^b^ relative to HLA-A2 D227K/T228A (Fig. 1*A*). In contrast, the agonist potency of FAT was only marginally enhanced in the context of HLA-A2 relative to HLA-A2 D227K/T228A and, consistent with the notion of an optimal activation window, decreased slightly in the context of HLA-A2 A245V/K^b^ relative to HLA-A2 (Fig. 1*A* and *SI Appendix*, Fig. S3*A*). As a consequence, the agonist potency of FAT relative to the agonist potency of ELA was reduced at higher pMHCI/CD8 affinities (Fig. 1*B*), and in three of four replicate experiments, ELA was the most potent ligand in the context of HLA-A2 and HLA-A2 A245V/K^b^ (Fig. 1 *B–E*).

**Fig. 2.**
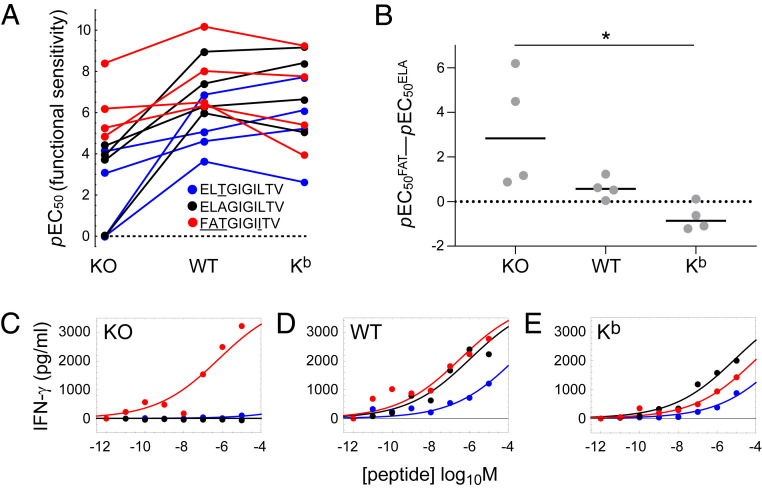
CD8 reorders the agonist hierarchy of peptide ligands that induce the production of IFN-γ. Clonal MEL5 CD8^+^ T cells were activated for 4 h with C1R cells expressing comparable levels of HLA-A2 D227K/T228A (KO), wild-type HLA-A2 (WT), or HLA-A2 K^b^ (K^b^) pulsed with various concentrations of 3T (blue), ELA (black), or FAT (red). Secretion of IFN-γ was measured using an enzyme-linked immunosorbent assay. (*A*) Functional sensitivity (*p*EC_50_) for each peptide ligand in the context of each MHCI. Four replicate experiments are shown. Values below the limit of estimation were set to zero for graphical purposes and treated as missing data for statistical purposes. *P* = 0.0042 for the ligand effect and *P* = 0.00069 for the MHCI effect (two-way ANOVA with Tukey’s post hoc test). (*B*) The agonist potency of FAT relative to the agonist potency of ELA expressed as *p*EC_50_^FAT^ − *p*EC_50_^ELA^, which is equivalent to the logarithm of the fold difference in functional sensitivity. Four replicate experiments are shown. Horizontal bars indicate median values. *P < 0.05 (one-way ANOVA with Tukey’s post hoc test). (*C–E*) Representative peptide titration experiment used to calculate the parameters in *A* and *B*. Curves were fitted in Mathematica. All four replicate experiments are shown in *SI Appendix*, Figs. S4 and S5.

To confirm these findings, we quantified the production of IFN-γ by clonal MEL5 CD8^+^ T cells in response to 3T, ELA, or FAT presented in the context of HLA-A2 D227K/T228A, wild-type HLA-A2, or HLA-A2 K^b^ (*SI Appendix*, Figs. S4 and S5). Functional sensitivity was again determined as the *p*EC_50_ value for each parameter combination (Fig. 2*A*). The activation data were largely analogous to those obtained with MEL5 TCR^+^ CD8^+^ J.RT3-T3.5 cells. In particular, the agonist potency of FAT was enhanced in the context of HLA-A2 relative to HLA-A2 D227K/T228A and decreased slightly in the context of HLA-A2 K^b^ relative to HLA-A2 (Fig. 2*A* and *SI Appendix*, Fig. S3*B*), mirroring the downturn in functional sensitivity observed with MEL5 TCR^+^ CD8^+^ J.RT3-T3.5 cells in the context of HLA-A2 A245V/K^b^ relative to HLA-A2 (Fig. 1*A* and *SI Appendix*, Fig. S3*A*). As a consequence, the agonist potency of FAT relative to the agonist potency of ELA was again reduced at higher pMHCI/CD8 affinities (Fig. 2*B*), and in three of four replicate experiments, ELA was the most potent ligand in the context of HLA-A2 K^b^ (Fig. 2 *B–E*).

In further experiments, we quantified the production of IFN-γ and MIP-1β by clonal MEL5 CD8^+^ T cells in response to ELA or 1I, a superagonist characterized for the purposes of this work, presented in the context of HLA-A2 D227K/T228A, wild-type HLA-A2, or HLA-A2 K^b^ (Table 1 and *SI Appendix*, Fig. S6). Irrespective of the functional readout, 1I was the more potent agonist in the context of HLA-A2 D227K/T228A and HLA-A2, whereas ELA was the more potent agonist in the context of HLA-A2 K^b^, thereby eliminating the possibility of a spurious link between the observed differential focusing effect and the anchor residue mutation present in FAT (*SI Appendix*, Fig. S6).

To confirm these findings in another intrinsically controlled system, we used a similar approach to determine the extent to which CD8 modulated functional responses initiated via the ILA1 TCR, which recognizes the human telomerase reverse transcriptase epitope ILAKFLHWL_540–548_ (ILA) restricted by HLA-A2. Biophysically defined peptide ligands, including a weak agonist (5Y), the wild-type epitope (ILA), and two superagonists (3G and 3G8R), were again selected for the purposes of this work to introduce a range of TCR/pMHCI affinities (*SI Appendix*, Table S1). Irrespective of the functional readout, the agonist potencies of 5Y and ILA were enhanced in the context of HLA-A2 and HLA-A2 K^b^ relative to HLA-A2 D227K/T228A, whereas the agonist potencies of 3G and 3G8R were only marginally enhanced in the context of HLA-A2 relative to HLA-A2 D227K/T228A and decreased slightly in the context of HLA-A2 K^b^ relative to HLA-A2 (*SI Appendix*, Figs. S7 and S8). As a consequence, ILA was the most potent ligand in the context of HLA-A2 K^b^, thereby validating the general applicability of differential focusing beyond the idiosyncratic properties of a singular TCR (*SI Appendix*, Figs. S7 and S8).

**Fig. 3.**
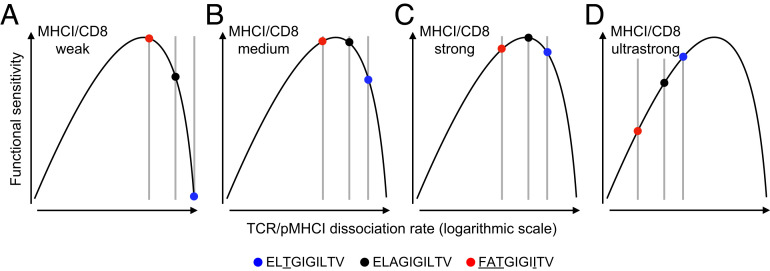
Theoretical interpretation of the differential focusing effect mediated by CD8. Graphical representation of the differential focusing effect based on two key assumptions: 1) functional sensitivity depends nonmonotonically on the TCR/pMHCI dissociation rate; and 2) the pMHCI/CD8 interaction affects the TCR/pMHCI dissociation rate by an invariant factor, equivalent to translation on a logarithmic scale (*SI Appendix*). (*A–C*) Modulation of the pMHCI/CD8 interaction moves peptide ligands along this curve, altering the agonist hierarchy as a function of the TCR/pMHCI dissociation rate. (*D*) A hypothetical ultrastrong pMHCI/CD8 interaction would be expected to reverse the agonist hierarchy from FAT > ELA > 3T to FAT < ELA < 3T.

Collectively, these results can be interpreted and understood in biological terms if two key assumptions are made: 1) functional sensitivity depends nonmonotonically on the TCR/pMHCI dissociation rate (13); and 2) the pMHCI/CD8 interaction affects the TCR/pMHCI dissociation rate by an invariant factor, equivalent to translation on a logarithmic scale (Fig. 3). In this scenario, ligands that are recognized poorly in the absence of a pMHCI/CD8 interaction become more potent in the presence of a physiological pMHCI/CD8 interaction and achieve optimal agonist potency in the presence of a supraphysiological pMHCI/CD8 interaction, whereas ligands that are recognized strongly in the absence of a pMHCI/CD8 interaction straddle an optimum in the presence of a physiological pMHCI/CD8 interaction and become less potent in the presence of a supraphysiological pMHCI/CD8 interaction. Accordingly, the agonist hierarchy of peptide ligands, which is dictated in isolation by the TCR/pMHCI interaction, can be reordered as a function of coengagement by CD8.

## Discussion

CD8^+^ T cells are inherently promiscuous and can recognize more than a million different peptide ligands via the TCR (23–26). It is well established that CD8 can enhance the functional sensitivity of antigen recognition, but in any given monoclonal system, it does not necessarily follow that CD8 will affect the agonist potency of every cognate ligand in a similar manner. Indeed, theoretical studies have suggested that the agonist hierarchy of peptide ligands can be modified or even reversed across a range of pMHCI/CD8 affinities, such that a differential focusing effect acts to optimize the recognition of particular ligands in the context of an individual TCR (9, 10, 15). Our data provide experimental confirmation of these predictions.

The biological relevance of differential focusing remains unknown, but hypothetical considerations suggest that such an effect may be advantageous in vivo, especially if accompanied by feedback mechanisms that enable the process of specificity adjustment to converge on a foreign antigen. Optimal recognition of a particular agonist in this manner would maximize immune efficacy during the process of clonal expansion and simultaneously minimize the risk of autoimmunity. Affinity maturation subserves an equivalent function in B cells. In more general terms, differential focusing also provides a solution to the “Mason paradox,” allowing a high degree of immune specificity alongside sufficient coverage of the peptide universe within a relatively small naive repertoire via the incorporation of degenerate TCRs (17).

Although it remains to be determined how differential focusing could operate in vivo and to what extent this might occur throughout the lifespan of any given clonotype, elegant studies have already provided important mechanistic clues. For example, double-positive thymocytes can transcriptionally down-regulate CD8 (27), and antigen encounters in the periphery can dynamically modulate clonal responsiveness via the selective internalization of CD8 (28). In addition, coreceptor use can be switched between the functionally distinct isoforms CD8-αα and CD8-αβ (29), which are further modifiable via glycosylation (30–32), and cytokine signals can transcriptionally alter the expression of CD8 (33). All of these processes affect the signaling threshold for activation via the TCR in a manner akin to affinity variation in the pMHCI/CD8 interaction (12, 34). Accordingly, functional sensitivity depends on the kinetics of signalosome development (9, 10), which is determined by agonist potency and regulated by CD8 (35).

In line with earlier theoretical predictions, the data presented here show that agonist potency, quantified in terms of functional sensitivity, can be differentially modulated across a range of TCR/pMHCI affinities by CD8. If this phenomenon occurs in vivo, as suggested by previous mechanistic studies, then immune reactivity could be focused on individual peptide ligands in the context of antigen-driven clonal expansions. On the basis of these collective observations, we propose that specificity adjustment operates at the level of individual clonotypes to safeguard the host in the face of an ongoing immune response, simultaneously facilitating the targeted delivery of effector functions and mitigating the risk of bystander damage, which can be triggered by inherently degenerate and therefore potentially autoreactive TCRs.

## Materials and Methods

### Cells.

MEL5 TCR^+^ CD8^+^ J.RT3-T3.5 cells were maintained in Roswell Park Memorial Institute (RPMI) 1640 medium containing 100 U/mL penicillin, 100 μg/mL streptomycin, 2 mM L-glutamine, and 10% heat-inactivated fetal calf serum (all from Thermo Fisher Scientific) (R10). Clonal MEL5 and ILA1 CD8^+^ T cells were maintained in R10 supplemented with 200 IU/mL IL-2 and 25 ng/mL IL-15 (both from PeproTech). The MEL5 TCR is specific for the heteroclitic HLA-A2–restricted Melan-A epitope ELA (18, 36), and the ILA1 TCR is specific for the HLA-A2–restricted human telomerase reverse transcriptase epitope ILA (37). Human embryonic kidney (HEK) 293 cells were grown in Dulbecco’s modified Eagle’s medium (Sigma-Aldrich) supplemented with 100 U/mL penicillin, 100 μg/mL streptomycin, 2 mM L-glutamine, 10% heat-inactivated fetal calf serum, and 10 mM HEPES (all from Thermo Fisher Scientific). C1R cells expressing comparable levels of HLA-A2 or variants thereof were generated and maintained as described previously (38).

### Peptides.

All peptides were synthesized at >95% purity using standard Fmoc chemistry (BioSynthesis Inc.).

### Lentiviruses.

The α and β chains of the MEL5 TCR were engineered to contain mouse constant domains (39) and cloned into a single pSF–Lenti–EF-1α lentiviral vector (Oxford Genetics) separated by a P2A sequence (Genewiz). The α and β chains of CD8 were cloned similarly into a single pSF–Lenti–EF-α lentiviral vector (Oxford Genetics) separated by an internal ribosomal entry site sequence (Genewiz). HEK 293 cells were cotransfected with the MEL5 TCR or CD8-αβ lentiviral vectors and the packaging plasmids pMDLg/pRRE, pRSV-Rev, and pCMV-VSV-G using Turbofect Transfection Reagent (Thermo Fisher Scientific). Lentiviral particles were concentrated using Lenti-X Concentrator (Takara Bio).

### Generation of MEL5 TCR^+^ CD8^+^ J.RT3-T3.5 Cells.

TCR-deficient J.RT3-T3.5 cells were transduced with MEL5 TCR lentiviral particles and magnetically enriched using anti-murine TCR-β–PE (clone REA318) in conjunction with anti-PE MicroBeads (Miltenyi Biotec). MEL5 TCR^+^ J.RT3-T3.5 cells were then transduced with CD8-αβ lentiviral particles, and MEL5 TCR^+^ CD8^+^ J.RT3-T3.5 cells were flow-purified using an Influx Cell Sorter (BD Biosciences).

### Quantification of Activation-Induced CD69.

C1R cells expressing comparable levels of HLA-A2 D227K/T228A, wild-type HLA-A2, or HLA-A2 A245V/K^b^ were pulsed for 1 h with various concentrations of the indicated peptides. Cells were then washed twice with RPMI 1640 medium containing 100 U/mL penicillin and 100 μg/mL streptomycin and resuspended in R10. Each assay included 1.5 × 10^5^ peptide-pulsed C1R cells and 5 × 10^4^ MEL5 TCR^+^ CD8^+^ J.RT3-T3.5 cells. Unpulsed targets were used as negative controls. Expression of CD69 on the surface of MEL5 TCR^+^ CD8^+^ J.RT3-T3.5 cells was measured after 6 h using the following directly conjugated monoclonal antibodies: anti-CD8-α–PE-Cy7 (clone RPA-T8; Thermo Fisher Scientific), anti-CD8-β–eFluor660 (clone SIDI8BEE; Thermo Fisher Scientific), anti-CD69–BV421 (clone FN50; BioLegend), and anti-HLA-A2–FITC (clone BB7.2; BioLegend). Nonviable cells were excluded from the analysis using LIVE/DEAD Fixable Aqua (Thermo Fisher Scientific). Data were acquired using a NovoCyte Flow Cytometer (ACEA Biosciences) and analyzed using FlowJo software version 10.6.1 (FlowJo LLC).

### Quantification of Activation-Induced IFN-γ and MIP-1β.

C1R cells expressing comparable levels of HLA-A2 D227K/T228A, wild-type HLA-A2, or HLA-A2 K^b^ were pulsed for 1 h with various concentrations of the indicated peptides. Cells were then washed twice with RPMI 1640 medium containing 100 U/mL penicillin and 100 μg/mL streptomycin and resuspended in R10. Each assay included 6 × 10^4^ peptide-pulsed C1R cells and 3 × 10^4^ clonal MEL5 or ILA1 CD8^+^ T cells. Unpulsed targets were used as negative controls. Supernatants were harvested after 4 h and evaluated for IFN-γ or MIP-1β using enzyme-linked immunosorbent assays (R&D Systems).

### Statistics.

Functional assay data were processed using simultaneous nonlinear least squares parameter estimation encoded in Mathematica (23). Functional sensitivity (*p*EC_50_) was expressed as the decimal cologarithm *p* of the 50% efficacy concentration (EC_50_). Assay-derived estimates of *p*EC_50_ were treated as input data for subsequent analyses of variance. Data were analyzed using a one-way or two-way ANOVA with Tukey’s post hoc test in Mathematica or Prism software version 8 (GraphPad).

## Supplementary Material

Supplementary File

## Data Availability

All study data are included in the article and/or *SI Appendix*.
