# Supplementary material for: CD8 coreceptor-mediated focusing can reorder the agonist hierarchy of peptide ligands recognized via the T cell receptor
Source: Proc Natl Acad Sci U S A. 2021 Jul 16;118(29):e2019639118. doi: 10.1073/pnas.2019639118 (PMC8307375; doi:10.1073/pnas.2019639118)
Supplement: Supplementary File [file pnas.2019639118.sapp.pdf]

SUPPORTING INFORMATION

CD8 coreceptor-mediated focusing can reorder the agonist hierarchy of peptide ligands recognized via the T cell receptor

Mathew Clement[1,*], Lea Knezevic[2,*], Tamsin Dockree[1], James E. McLaren[1], Kristin Ladell[1], Kelly L. Miners[1], Sian Llewellyn-Lacey[1], Anzelika Rubina[1], Ore Francis[2], David K. Cole[3], Andrew K. Sewell[1,4], John S. Bridgeman[5], David A. Price[1,4], Hugo A. van den Berg[6,*], Linda Wooldridge[2,*]

[1]Division of Infection and Immunity, Cardiff University School of Medicine, University Hospital of Wales, Cardiff CF14 4XN, UK.

[2]Faculty of Health Sciences, University of Bristol, Biomedical Sciences Building, Bristol BS8 1TD, UK.

[3]Immunocore Ltd., 92 Park Drive, Abingdon OX14 4RY, UK.

[4]Systems Immunity Research Institute, Cardiff University School of Medicine, University Hospital of Wales, Cardiff CF14 4XN, UK.

[5]Immetacyte Ltd, 48 Grafton Street, Manchester M13 9XX, UK.

[6]Warwick Mathematics Institute, University of Warwick, Coventry CV4 7AL, UK.

Correspondence: Professor Linda Wooldridge, Faculty of Health Sciences, University of Bristol, Biomedical Sciences Building, Bristol BS8 1TD, UK.

E-mail: linda.wooldridge@bristol.ac.uk.

*These authors contributed equally to this work.

This PDF file includes:

- **Supplementary Methods**
- **Supplementary Table S1**
- **Supplementary Figures S1 to S8**
- **Supplementary Mathematical & Statistical Considerations**
- **Supplementary References**

SUPPLEMENTARY METHODS

Protein production and surface plasmon resonance

TCRs and biotinylated pMHCI molecules were refolded and purified as described previously (1). Surface plasmon resonance analysis was performed using a BIAcore 3000 (GE Healthcare). Biotinylated pMHCI proteins (200–500 response units) were immobilized to streptavidin, which was chemically linked to the surface of a CM5 sensor chip. For equilibrium analyses, ten serial dilutions of each TCR were injected over the surface of the sensor chip at 25°C. Results were analyzed using BIAevaluation version 3.1 (GE Healthcare), Excel (Microsoft), and Origin version 6.1 (OriginLab). Equilibrium binding constant (K_D) values were calculated using a nonlinear curve fit (y = [P1x] / [P2+x]).

Supplementary Table S1. TCR/pMHCI dissociation constants and kinetics for agonists of the ILA1 TCR.

MHCI	Epitope	k_{on} ($M^{-1}s^{-1}$)	k_{off} (s^{-1})	K_D (μM)
HLA-A*0201	ILAK**<u>Y</u>**LHWL (5Y)	1.3×10^3	0.32	242 ± 20 (2)
HLA-A*0201	ILAKFLHWL (ILA)	4.5×10^3	0.15	34 ± 2 (2)
HLA-A*0201	IL**<u>G</u>**KFLHWL (3G)	1.6×10^4	0.05	3.7 ± 0.2 (2)
HLA-A*0201	IL**<u>G</u>**KFLH**<u>R</u>**L (3G8R)	3.0×10^4	0.16	1.0 ± 0.1 (2)*

*Remeasured here as k_{on} = 9.5 x 10^3 $M^{-1}s^{-1}$, k_{off} = 0.098 s^{-1}, and K_D = 10.3 μM.

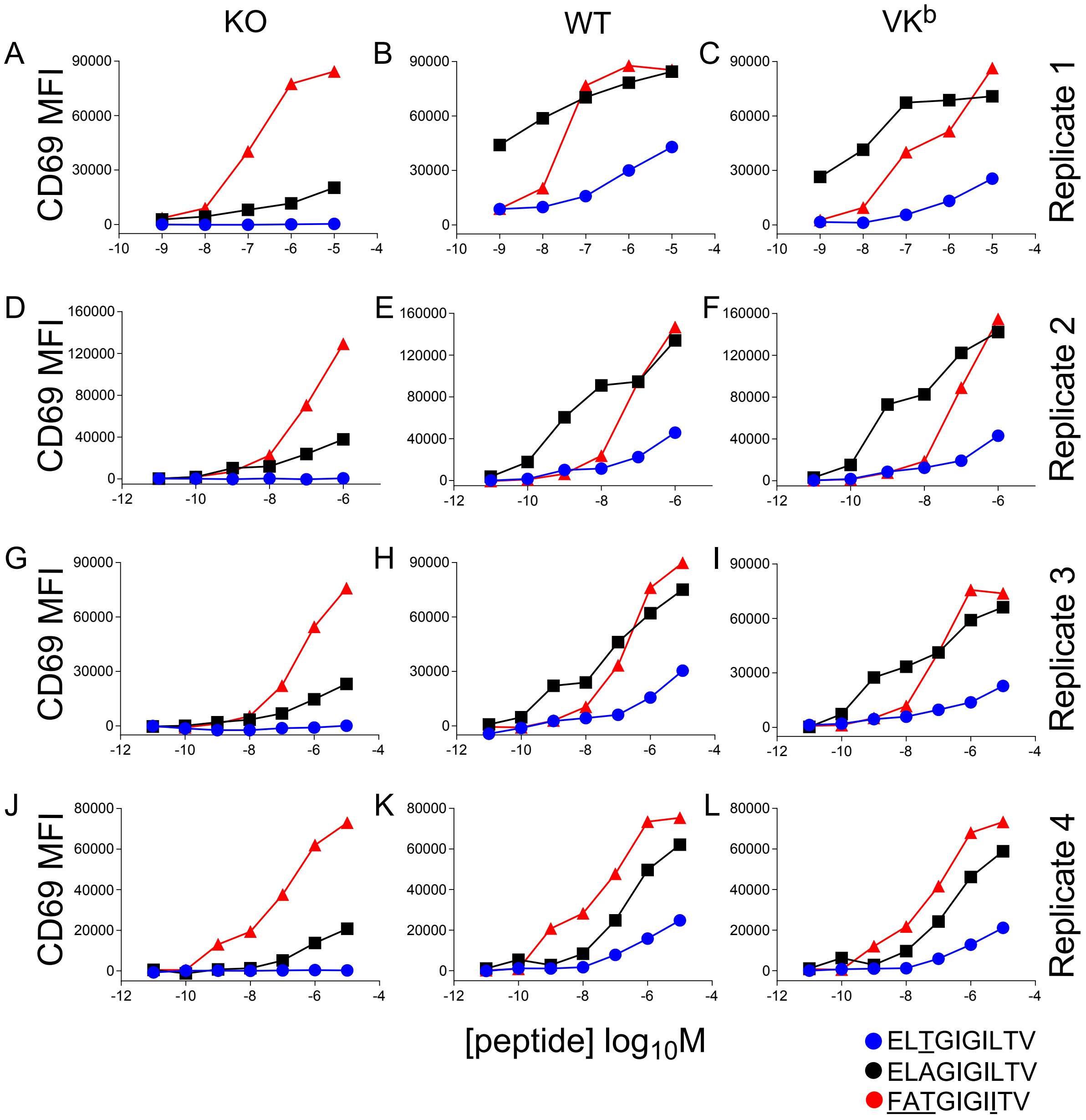

Supplementary Figure S1. CD8 reorders the agonist hierarchy of peptide ligands that induce the expression of CD69 via the MEL5 TCR. MEL5 TCR$^+$ CD8$^+$ J.RT3-T3.5 cells were activated for 6 h with C1R cells expressing comparable levels of HLA-A2 D227K/T228A (KO), wildtype HLA-A2 (WT), or HLA-A2 A245V/K^b (VK^b) pulsed with various concentrations of 3T (blue), ELA (black), or FAT (red). Surface expression of CD69 was measured via flow cytometry. (**A**–**C**) Experimental replicate 1. (**D**–**F**) Experimental replicate 2. (**G**–**I**) Experimental replicate 3. (**J**–**L**) Experimental replicate 4.

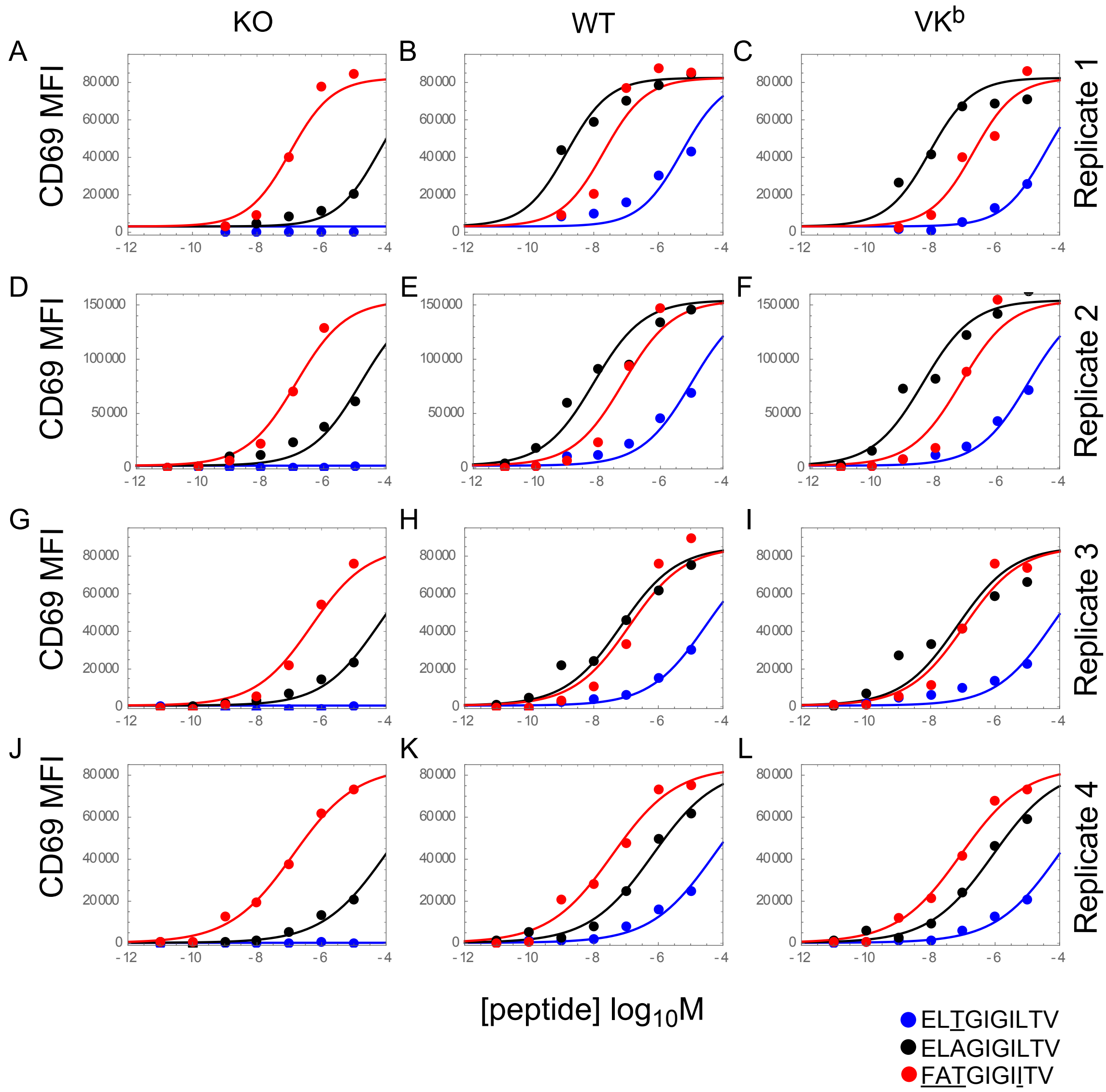

Supplementary Figure S2. CD8 reorders the agonist hierarchy of peptide ligands that induce the expression of CD69 via the MEL5 TCR. Experimental details as in Supplementary Figure S1. Curves were fitted to the same data in *Mathematica*.

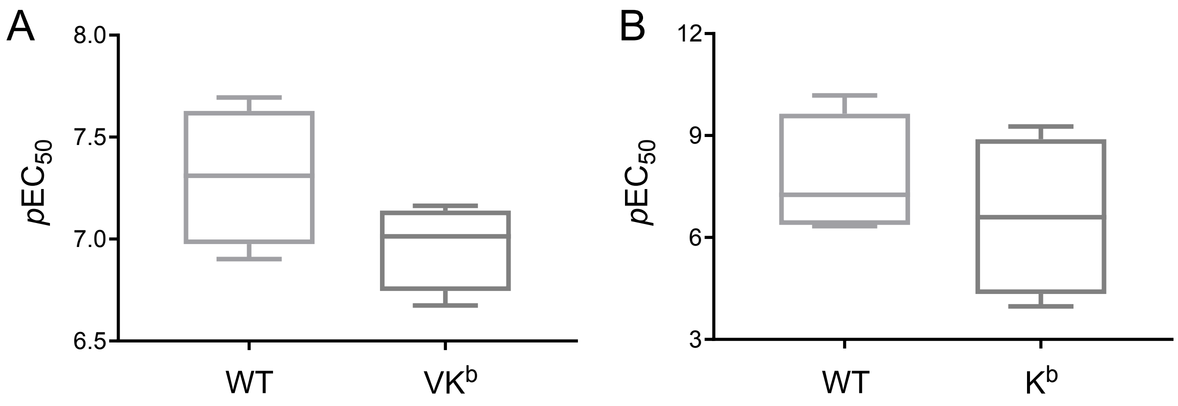

Supplementary Figure S3. Enhanced coreceptor interactions reduce the potency of a strong agonist recognized via the MEL5 TCR. (**A**) Box and whisker plots summarizing the data shown in Figure 1A for FAT. (**B**) Box and whisker plots summarizing the data shown in Figure 2A for FAT.

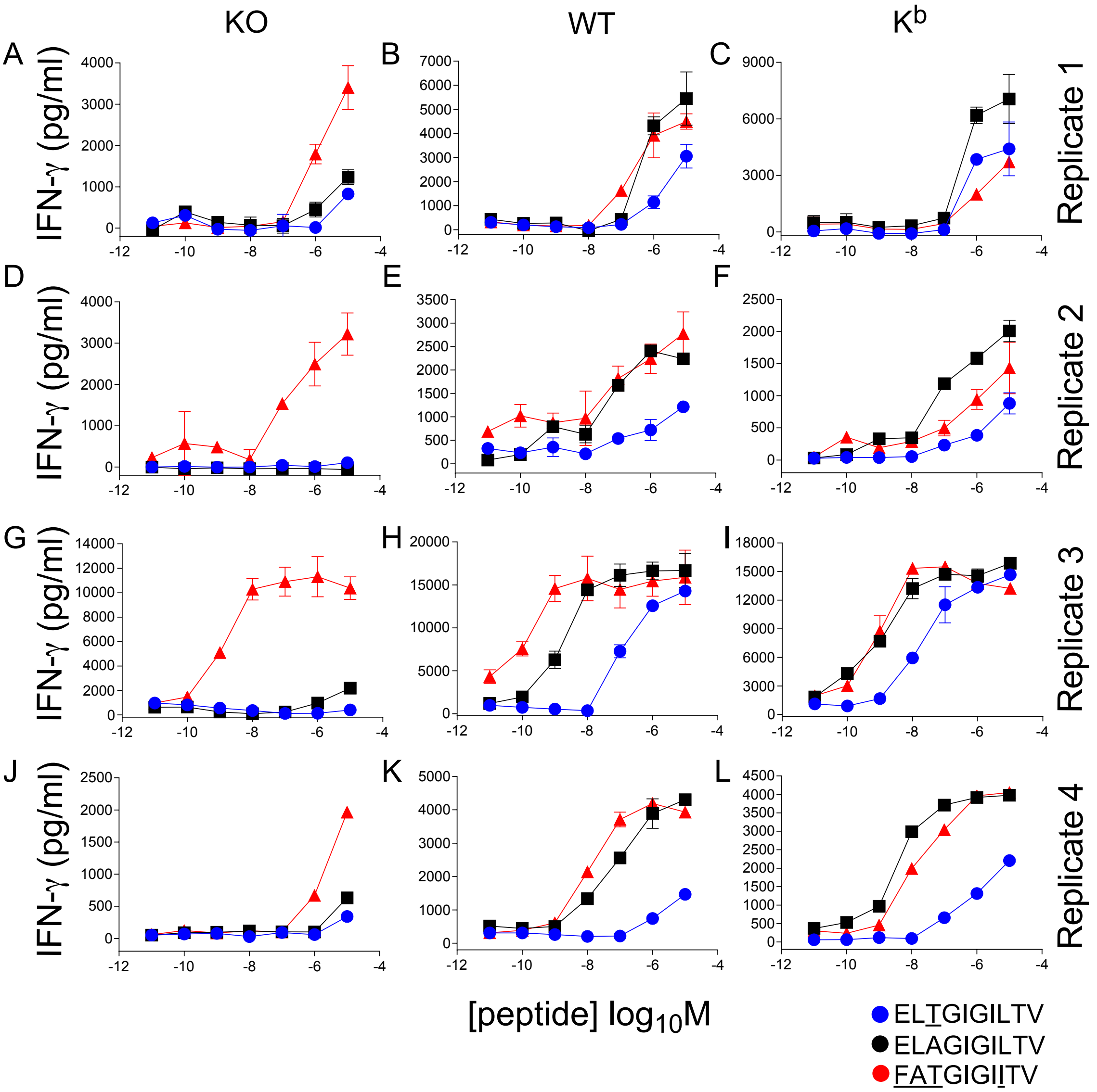

Supplementary Figure S4. CD8 reorders the agonist hierarchy of peptide ligands that induce the production of IFN-γ via the MEL5 TCR. Clonal MEL5 CD8$^+$ T cells were activated for 4 h with C1R cells expressing comparable levels of HLA-A2 D227K/T228A (KO), wildtype HLA-A2 (WT), or HLA-A2 K^b (K^b) pulsed with various concentrations of 3T (blue), ELA (black), or FAT (red). Secretion of IFN-γ was measured via ELISA. (**A–C**) Experimental replicate 1. (**D–F**) Experimental replicate 2. (**G–I**) Experimental replicate 3. (**J–L**) Experimental replicate 4. Each data point represents the mean of duplicate measurements. Error bars show SD.

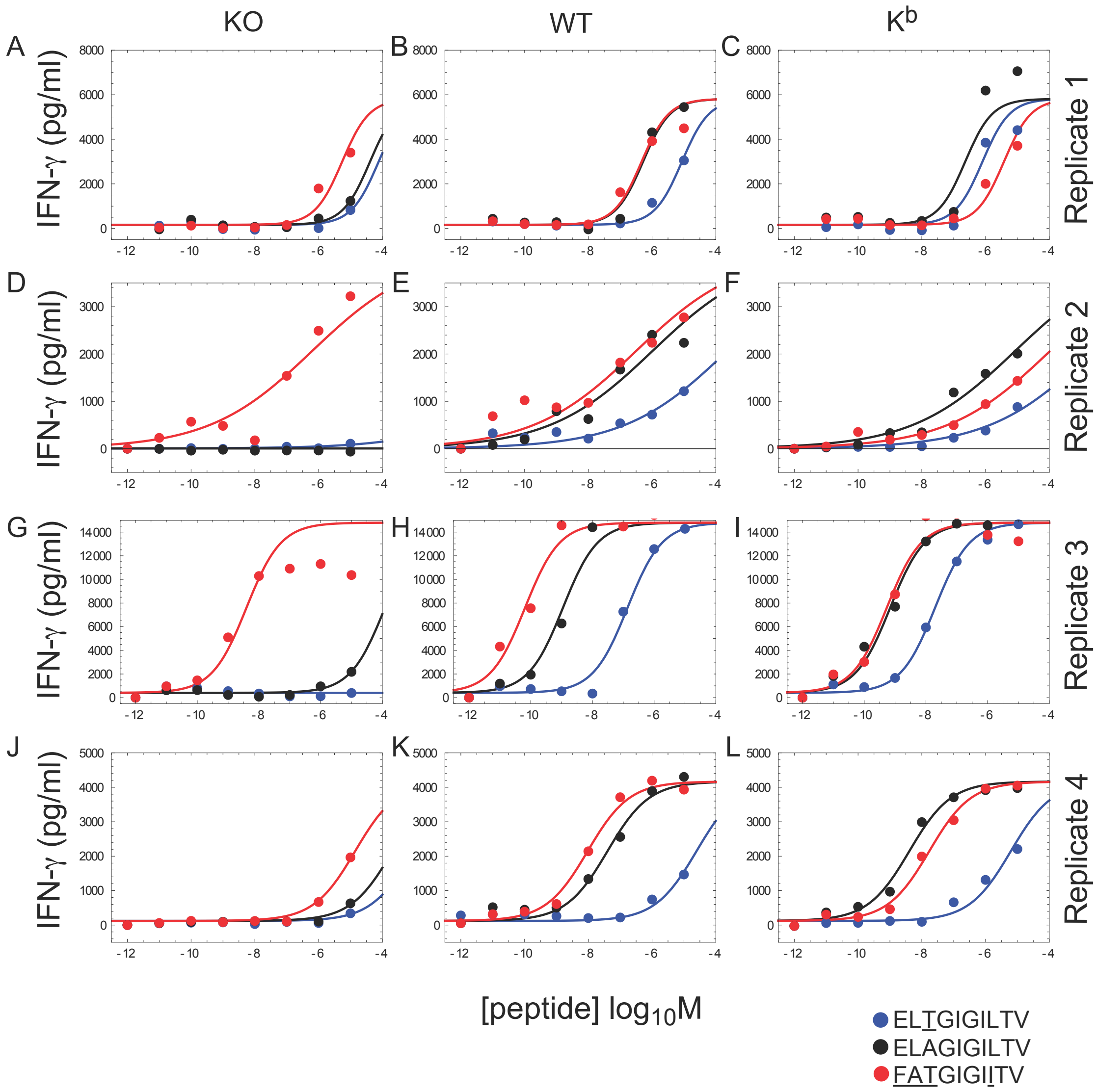

Supplementary Figure S5. CD8 reorders the agonist hierarchy of peptide ligands that induce the production of IFN-γ via the MEL5 TCR. Experimental details as in Supplementary Figure S4. Curves were fitted to the same data in *Mathematica*.

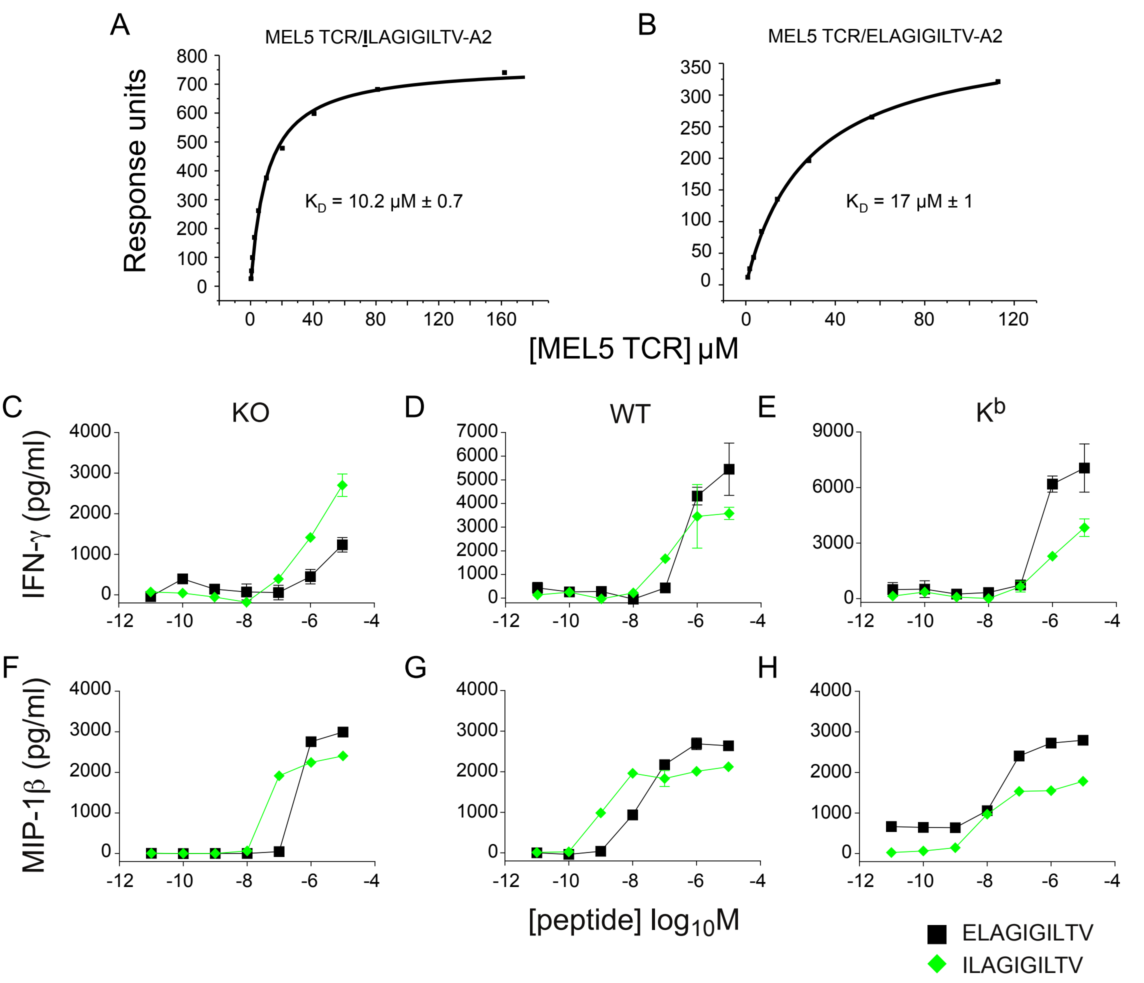

Supplementary Figure S6. CD8 reorders the agonist hierarchy of peptide ligands that induce the production of IFN-γ and MIP1-β via the MEL5 TCR. (**A**, **B**) Surface plasmon resonance curves showing equilibrium binding of HLA-A2 complexed with ILAGIGILTV (A) or ELAGIGILTV (B) to the MEL5 TCR. (**C–H**) Clonal MEL5 $CD8^+$ T cells were activated for 4 h with C1R cells expressing comparable levels of HLA-A2 D227K/T228A (KO), wildtype HLA-A2 (WT), or HLA-A2 K^b (K^b) pulsed with various concentrations of ELA (black) or 1I (green). Secretion of IFN-γ (C–E) or MIP-1β (F–H) was measured via ELISA. Each data point represents the mean of duplicate measurements. Error bars show SD.

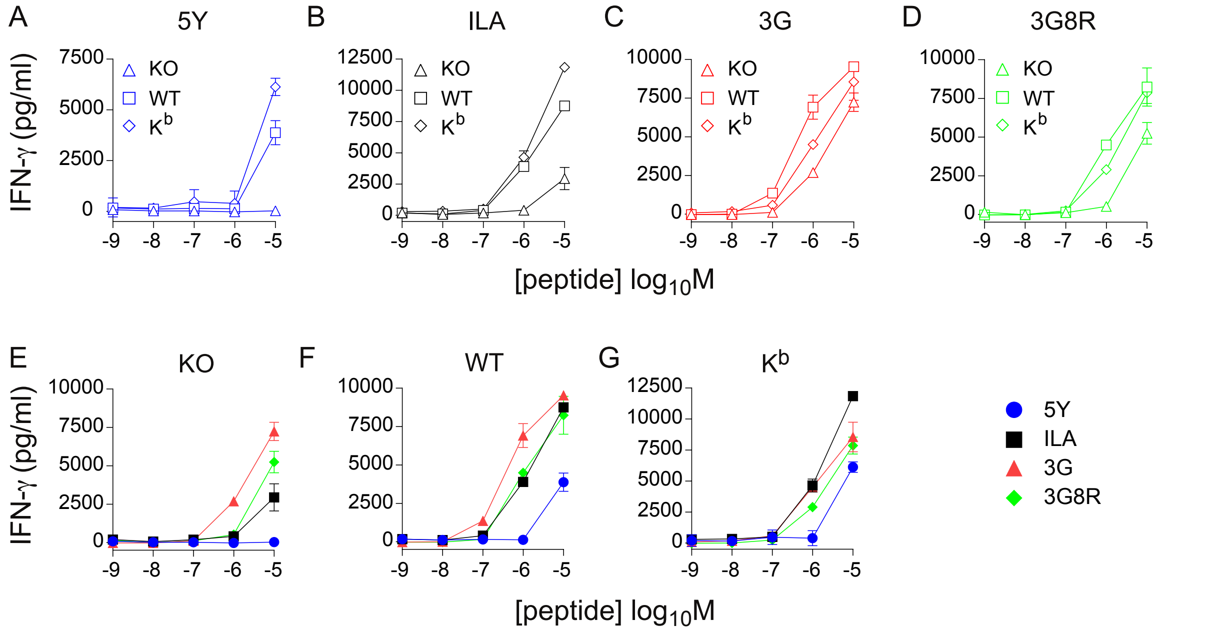

Supplementary Figure S7. CD8 reorders the agonist hierarchy of peptide ligands that induce the production of IFN-γ via the ILA1 TCR. Clonal ILA1 CD8$^+$ T cells were activated for 4 h with C1R cells expressing comparable levels of HLA-A2 D227K/T228A (KO), wildtype HLA-A2 (WT), or HLA-A2 K^b (K^b) pulsed with various concentrations of 5Y (blue), ILA (black), 3G (red), or 3G8R (green). Secretion of IFN-γ was measured via ELISA. (**A**–**D**) Data are shown for each peptide. (**E**–**G**) Data are shown for each target. Each data point represents the mean of duplicate measurements. Error bars show SD.

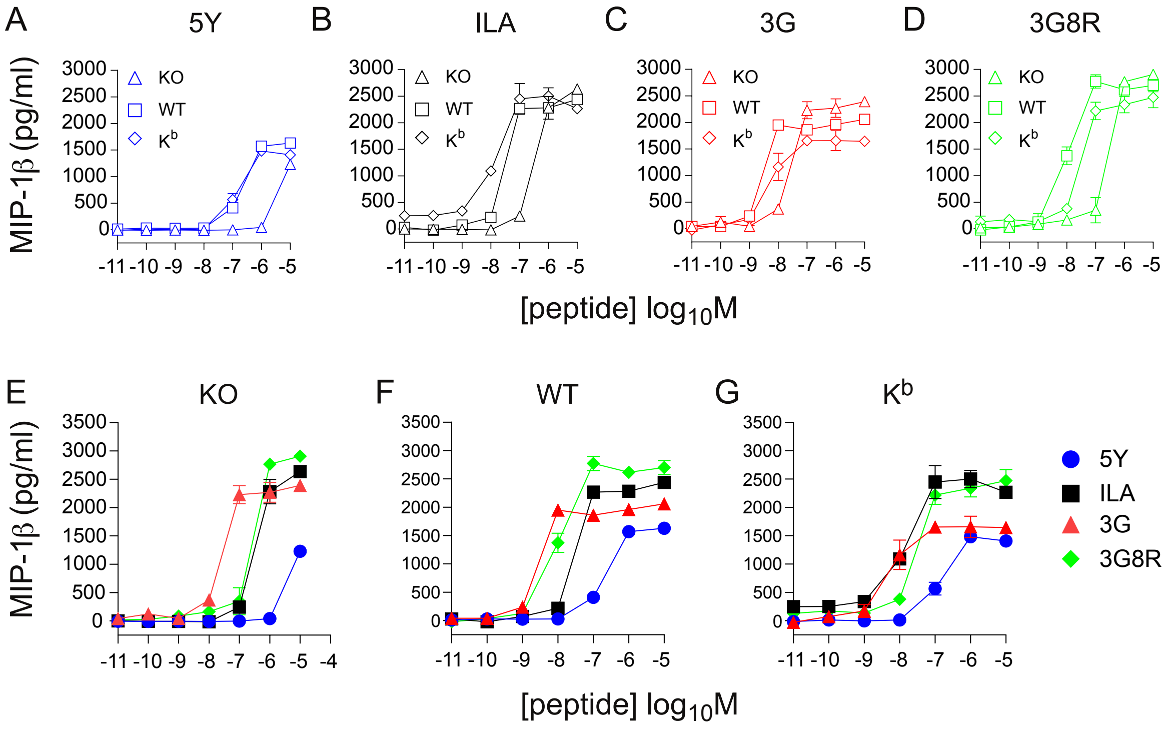

Supplementary Figure S8. CD8 reorders the agonist hierarchy of peptide ligands that induce the production of MIP-1β via the ILA1 TCR. Clonal ILA1 $CD8^+$ T cells were activated for 4 h with C1R cells expressing comparable levels of HLA-A2 D227K/T228A (KO), wildtype HLA-A2 (WT), or HLA-A2 K^b (K^b) pulsed with various concentrations of 5Y (blue), ILA (black), 3G (red), or 3G8R (green). Secretion of MIP-1β was measured via ELISA. (**A**–**D**) Data are shown for each peptide. (**E**–**G**) Data are shown for each target. Each data point represents the mean of duplicate measurements. Error bars show SD.

SUPPLEMENTARY MATHEMATICAL & STATISTICAL CONSIDERATIONS

Introduction

The mechanism by which the hierarchy of ligand potency with respect to a given TCR clonotype is reordered depends in essence on the propositions that: (i) the dependence of functional sensitivity on the TCR/pMHCI dissociation rate is non-monotone (*i.e.*, there is an optimum dissociation rate value, such that the clonotype at hand is most responsive to ligands exhibiting this value); and (ii) the pMHCI/CD8 interaction results in a diminishment of this dissociation rate. If one is prepared to accept (i) and (ii) as empirical facts attested in the experimental literature, then the "graphical proof" as given in the main text suffices (Figure 3). Here, we delve somewhat deeper into the background of these two fundamental propositions (3). We should perhaps emphasize from the outset that the focusing mechanism depends solely on (i) and (ii), and that any more specific assumptions are made only by way of further explication, leaving open the possibility of alternative detailed models underlying (i) and (ii) and, by extension, the focusing mechanism.

The CD8 boost

We begin by restating proposition (i) more formally. Let λ denote the TCR/pMHCI dissociation rate and λ_0 the optimal value, for which the functional sensitivity $p\mathrm{EC}_{50}$ attains its maximum value, and define $x = \ln\{\lambda/\lambda_0\}$. A Taylor expansion about this maximum may be written as follows:

(*) $$p\mathrm{EC}_{50}(x) = p\mathrm{EC}_{50}^{\circ} - c_2x^2 + c_3x^3 + c_4x^4 + \cdots,$$

where $c_2 > 0$ and the subsequent $c_3, c_4, \ldots$ may be of either sign, and $p\mathrm{EC}_{50}^{\circ}$ is the $p\mathrm{EC}_{50}$ at $x = 0$ (*i.e.*, the optimum). Retaining only the leading terms to second order[1], let us consider the change in $p\mathrm{EC}_{50}$ when x is diminished by a certain amount ξ, as per proposition (ii). Thus, $x \to x - \xi$ with $\xi > 0$. We find that:

---

[1]The truncations to second order in (*) and (**) are not essential, but working only to second-order, we are able to exhibit the main qualitative features of the theory through particularly simple formulae. Inclusion of higher-order terms results in lengthier, more cumbersome expressions and requires a numerical approach, which although straightforward, entails technical intricacies that we propose to leave aside for the present purposes.

$$\Delta p\text{EC}_{50} = 2c_2\xi(x - \xi/2),$$

which shows that $\Delta p\text{EC}_{50}$ is negative when $x = 0$. In other words, a ligand that is optimal before CD8 enhancement is applied becomes less optimal, whereas a ligand that is initially suboptimal at $x = \xi$ becomes optimal (with $\Delta p\text{EC}_{50} = c_2\xi^2$) and a ligand initially at $x = \xi/2$ does not change ($\Delta p\text{EC}_{50} = 0$). More generally, only ligands that are initially suboptimal (with $x > \xi/2$) experience an enhancement in functional sensitivity. Reordering of the ligand sensitivity hierarchy occurs when the differences in $p\text{EC}_{50}$ change sign as the CD8 effect is applied, which requires:

(Con) $$0 < x_1 + x_2 < \xi/2,$$

where x_1 and x_2 are the values before enhancement. When both x_1 and x_2 are negative, such that the ligands are *heteroclitic* as defined in reference (4), reordering will never occur, and when both are positive, as is typically the case, reordering will only occur if the ligands are sufficiently close to optimal initially, with the magnitude of the effect (ξ) governing the susceptible range. If we take the higher-order terms into account, the expressions become more involved, but the essential qualitative behavior remains the same.

Uniformity of the CD8 boost

We have assumed that all ligands are modified by an equal amount ξ. However, it is possible that different ligands are affected differently, and one obtains hierarchy reordering more readily assuming different values for ξ. The conservative choice is therefore to focus on the case where ξ is the same for all ligands. TCR/pMHCI interactions will often be discussed in terms of being more or less strongly dependent on the pMHCI/CD8 interaction, which could be taken to indicate differences in ξ, but such apparent variations in "CD8 dependence" will occur even when ξ is the same for all ligands. The reason is that for very weak ligands, we may consider the limiting case $x \gg \xi$, in which $\Delta p\text{EC}_{50} \approx 2c_2\xi x$ (*i.e.*, weak ligands receive a boost proportional to x, which essentially measures how weak they are), whereas for strong suboptimal ligands ($x \approx \xi/2$), only a weak (if any) CD8 effect is observed, and for near-optimal ligands ($x \approx 0$), we find that $\Delta p\text{EC}_{50} \approx -c_2\xi^2$ (*i.e.*, negative but smaller in magnitude compared with weak ligands).

TCR susceptibility to CD8 enhancement

A related but distinct question is whether ξ can be considered to have a universal value across the TCR repertoire. The answer to this question has no direct bearing on the main message of the present paper. It is nonetheless interesting to hypothesize that some TCRs may have greater ξ values than others, and that this parameter may then be regarded as expressing a TCR's *susceptibility* to CD8 enhancement. A small ξ means that weak ligands receive a less pronounced coreceptor boost and, moreover, the range of ligands that may exhibit potency reordering will be more restricted (recall the condition $0 < x_1 + x_2 < \xi/2$ derived above). On the other hand, a TCR with a relatively large ξ will be more responsive to the "CD8 assist", and reordering of the agonist hierarchy may occur among a larger set of potential ligands.

Focusable ligands vis-à-vis the peptide universe

Potency reordering happens for a restricted set of ligands that exist in the neighborhood of the optimum[2]. It is also within this restricted set that one may discuss the effect in terms of "focusing", which is the placement of different peptides on the optimum, as a function of the CD8 boost. This set is likely to be small relative to the size of the peptide universe. If we divide the size of the peptide universe by the size of the TCR repertoire, we arrive at the size of the set of peptides that each TCR would have to cover in order for the coverage to be complete. Taking 20^9 for nonapeptides and 10^6 for TCR repertoire size, the target number is found to be approximately half a million, which is of a magnitude comparable to that reported previously (5). This may perhaps be a crude way of estimating the cardinality of the set of potentially potent ligands associated with each TCR clonotype, and further refinements of this argument have been published elsewhere (6–8). These numbers show how appealing to intuition can be misleading: "half a million ligands" suggests a promiscuous TCR, whereas "one in a million" suggests the opposite. Yet both are true. One-to-one specificity, such that each TCR recognizes only a single peptide, would not be possible[3], whereas on the other hand, a considerable degree of specificity must clearly exist in order for the adaptive immune system to target pathogens and to avoid autoimmunity.

---

[2]This is not entirely correct. Condition (Con) may be satisfied by a pair consisting of a weak agonist and a weak heteroclitic peptide, the latter becoming even weaker as the former is boosted by the CD8 shift. However, as we argue below, such heteroclitic peptides are exceedingly rare, whereas weak agonists are quite common. Thus, whereas one may formally still regard this as "reordering of the potency hierarchy," the physiological interest of such focusing lies mainly with the more select group of near-optimal agonists.

Null ligands dissociate faster

We consider the statistical distribution of dissociation rates across the peptide universe for a fixed clonotype. In particular, we choose a peptide at random, let its dissociation rate be $\underline{\lambda}$, and let $F(x)$ denote the probability that $\ln\{\underline{\lambda}/\lambda_0\}$ does not exceed x (strictly speaking, we should write $F_i(x)$ here to indicate that this function depends on TCR clonotype i and may be different for different TCRs). Let us define:

$$\overline{x} = \int_{-\infty}^{+\infty} x dF(x) \text{ and } \sigma^2 = \int_{-\infty}^{+\infty} (x - \overline{x})^2 dF(x).$$

If $\overline{x} \approx 0$, we should find that most ligands are close to optimal. However, we must rule out this scenario, because each TCR would then recognize virtually every peptide, and we have seen that TCRs are not entirely nonspecific (even if promiscuous). If $\overline{x} < 0$, we should find that most peptides dissociate more slowly than optimal (heteroclitic) peptides. This outcome would not in principle hamper TCR/pMHCI recognition or specificity, but it would limit the number of pMHCI molecules that a given TCR molecule could scan in a given amount of time. Thus, we conclude that $\overline{x} > 0$, which is supported by the empirical evidence. It is well known that weak and ultraweak ("null") ligands dissociate *faster* than optimal ligands, and we surmise that the TCR effectively uses a mechanism akin to kinetic proofreading to measure the interaction time.

***p*EC50 probability distribution**

We have been dealing with "low-probability" events located in the tail of the distribution F. In such a setting, the probability that a peptide chosen at random satisfies $\ln\{\underline{\lambda}/\lambda_0\} < \xi$ can be estimated using the exponential overbound (a form of the Chebyshev inequality). We take the Maclaurin expansion of the cumulant generating function to second order, as is appropriate for moderate deviations[3]:

---

[3]As with (*) below, taking the second-order approximation in (**) results in simple expressions that convey the main qualitative features of the theory. In this case, inclusion of higher-order terms requires the determination, for a given TCR, of the dissociation rates for a large number of peptide ligands, such that their statistics can be determined to a high degree of accuracy. If this distribution were found to be log-normal (which is in fact to be expected on the basis of more general biophysical grounds), the second-order approximation in (**) would be exact.

(**) $$\ln\mathbb{E}[\exp\{t\ln\{\lambda/\lambda_0\}\}] \approx \bar{x}t + \sigma^2 t^2/2 \,.$$

We then obtain the following exponential overbound[4]:

(ExpO) $$\mathbb{P}[\ln\{\lambda/\lambda_0\} < \xi] \leq \exp\{-(\xi - \bar{x})^2/\sigma^2\},$$

which estimates the size of the set of potential "focusable" ligands for a given TCR in terms of the parameters ξ, $\bar{x}$, and σ^2 (the latter two parameters govern the TCR's degeneracy). As regards agonists satisfying $x > \xi/2$, these only experience a CD8 boost, without reordering of potency. The bulk of a TCR's weak agonists falls in this category. By the Large Deviations principle, we have the estimate[5]:

(LD) $$\mathbb{P}[p\mathrm{EC}_{50} \approx p\mathrm{EC}^{\circ}_{50} - \omega] \leq \exp\left\{-\left(\bar{x} - \sqrt{\omega/c_2}\right)^2/\sigma^2\right\},$$

which expresses the degeneracy of the TCR at hand at functional sensitivity $p\mathrm{EC}^{\circ}_{50} - \omega$. Incorporating the CD8 effect ξ into this formula, we obtain:

(LDbis) $$\ln\mathbb{P}[p\mathrm{EC}_{50} \approx p\mathrm{EC}^{\circ}_{50} - \omega; \xi] \leq -\left(\bar{x} - \xi - \sqrt{\omega/c_2}\right)^2/\sigma^2,$$

where we have used the "$\ln\mathbb{P}$" notation to emphasize that our primary concern is to estimate orders of magnitude. We are particularly interested in the effect of CD8 at a given functional sensitivity point ω (*i.e.*, how much more likely, due to CD8, the TCR is to have an agonist with functional sensitivity $p\mathrm{EC}_{50} = p\mathrm{EC}^{\circ}_{50} - \omega$):

---

[4]The expression on the righthand side should not be confused with the formula for the probability density of a Gaussian. However, there is a connection with the normal distribution by way of the truncation to second order that was made in eqn (**). There are various excellent texts on Large Deviations theory (9, 10).

[5]This expression can be further refined by considering "tilted" distributions, resulting in an improved approximation in the neighborhood of the mean of *F*. However, this region corresponds to ultraweak agonists and is therefore of less relevance. The formula also ignores a correction that needs to be made for heteroclitic agonists corresponding to lower $p\mathrm{EC}_{50}$ values. However, the Large Deviations principle assures us that probability mass is exponentially concentrated near the point of interest, which implies that this correction is unimportant unless ω is very close to zero.

$$\Delta_{\omega,\xi}\ln\mathbb{P} \approx \left(\xi^2 + 2\xi\left(\sqrt{\omega/c_2} - \overline{x}\right)\right)/\sigma^2.$$

Further developments

In summary, some key features of the ligand repertoire of a given TCR can be understood on the basis of posits (i) and (ii), along with the general principles of Large Deviations. Our group has made attempts to reconstruct, empirically, the probability distribution of the functional sensitivities across possible ligands for a defined TCR clonotype, employing an importance sampling technique (5), yielding a good agreement with eqn (LD). The ligand repertoires pertaining to individual TCRs can be aggregated to yield the systemic T cell repertoire. Similarly, we can further develop the theory to describe *antigen-presentation profiles*, which are the repertoires of peptides (both self and foreign) presented on healthy and unhealthy body cells and professional antigen-presenting cells in both primary and secondary lymphoid tissues, to account for the way the naive repertoire is created and how it changes in response to pathogenic challenges. We shall not pursue these further developments in more detail here, except to remark that the tools of Large Deviations again allow us to gain considerable insights departing from a minimal set of assumptions.

CD8 effect via modulation of the dissociation energy barrier

We next take up the question of the plausibility of propositions (i) and (ii). We shall begin with the latter posit. Consider the Arrhenius equation for the TCR/pMHCI dissociation rate[6]:

(Arrh) $$\lambda = \lambda^*\exp\{-U_a/k_BT\},$$

where λ^* is a prefactor with the same units as λ, k_B is the Boltzmann constant, and T is the absolute temperature, whereas U_a is the activation energy for the dissociation step in Boltzmann units. If we assume that the pMHCI/CD8 interaction adds a term of fixed magnitude to this energy barrier, U_{CD8}, then the CD8 boost decreases the dissociation rate by a constant factor, so that $\ln\{\lambda/\lambda_0\}$ is diminished by a fixed term, $\xi = U_{\text{CD8}}/k_BT$. It may be objected that U_{CD8} could take on different values for different ligands, in which case hierarchy reordering and focusing become trivially apparent. However, our main message in

---

[6]An excellent derivation of the Arrhenius equation from the perspective of modern mathematical statistics is given in reference (11).

this work is to explain that these phenomena do not critically depend on such heterogeneous CD8 effects, and that they will occur even if all ligands are susceptible to the common shift ξ.

TCR triggering rate dependence on off-rate

Empirical evidence in support of proposition (i) is well known, although the role of dissociation rate as the prime governor of functional sensitivity is contested by those who perceive an important role for the dissociation constant K_D ($K_D = \lambda/\alpha$, where α is the association rate, so there are only two independent factors among K_D, α, and λ; K_D is also called the "affinity" constant, but strictly speaking, this is $K_A = K_D^{-1} = \alpha/\lambda$). According to the following equation, both have an important role to play:

(Trig) $$W = \frac{\lambda \exp\{-\lambda/\lambda_R\}}{2}(K_D + R_T + Z_T)\left(1 - \sqrt{1 - \frac{4R_T Z_T}{K_D + R_T + Z_T}}\right),$$

where W is the rate at which the TCR is triggered during an interaction between a T cell and an antigen-presenting cell, λ_R is a rate parameter expressing the rate at which the TCR/CD3 complex reaches signalosome competence, R_T is the density of TCR molecules in the interaction area between the cells, and Z_T is the density of pMHCI molecules carrying the ligand of interest in that same area. Eqn (Trig) can be derived from basic mass-action kinetics of the TCR and pMHCI molecules[7]. The role of K_D is as follows. If $K_D \gg max\{R_T, Z_T\}$, then eqn (Trig) reduces to:

(Trig[a]) $$W = \alpha R_T Z_T \exp\{-\lambda/\lambda_R\},$$

whereas if $K_D \ll R_T$, eqn (Trig) reduces to:

(Trig[b]) $$W = \lambda Z_T \exp\{-\lambda/\lambda_R\},$$

and finally, if $K_D \ll Z_T$, eqn (Trig) reduces to:

(Trig[c]) $$W = \lambda R_T \exp\{-\lambda/\lambda_R\}.$$

[7]A full derivation of eqn (Trig) is given in reference (12). The formula in eqn (Trig) treats all ligands on the antigen-presenting cell as true null ligands, except the ligand of interest. For a more complete treatment, which allows that some, and possibly many, of the presented peptides may not be null, see (3).

The factor $\lambda\exp\{-\lambda/\lambda_R\}$ is non-monotone in λ and attains a maximum at $\lambda = \lambda_R$. It is thus apparent that, provided we treat W as a close correlate of $p\text{EC}_{50}$, we have a relationship underpinning proposition (i), with $\lambda_0 = \lambda_R$. We thus write $w = \lambda\exp\{-\lambda/\lambda_0\}$ and consider the following Taylor expansion for $\ln\{w/\lambda_0\}$ as a function of $x = \ln\{\lambda/\lambda_0\}$, about the point $\lambda = \lambda_0$:

$$\ln\{w/\lambda_0\} = -1 - \frac{x^2}{2} - \frac{x^3}{3!} - \frac{x^4}{4!} - \frac{x^5}{5!} - \cdots,$$

and therefore, if we assume[8] $p\text{EC}_{50} = \kappa + \gamma\ln\{w/\lambda_0\}$, we obtain:

$$p\text{EC}_{50} = \kappa - \gamma - \frac{\gamma}{2}x^2 - \frac{\gamma}{3!}x^3 - \cdots,$$

in agreement with eqn (*). Writing $p\text{EC}_{50} = p\text{EC}^\circ_{50} - \omega$, we have:

$$\omega = p\text{EC}^\circ_{50}\left(\frac{x^2}{2} + \frac{x^3}{6} + \frac{x^4}{24} + \cdots\right),$$

and this series may be inverted to give the non-heteroclitic value of $x = \ln\{\lambda/\lambda_0\}$:

$$x = \sqrt{\frac{\omega}{p\text{EC}^\circ_{50}/2}} - \frac{\omega}{3p\text{EC}^\circ_{50}} + \frac{1}{9\sqrt{2}}\left(\frac{\omega}{p\text{EC}^\circ_{50}}\right)^{3/2} - \frac{2}{135}\left(\frac{\omega}{p\text{EC}^\circ_{50}}\right)^2 + \cdots,$$

of which only the first term appears in eqn (LD), with $c_2 = p\text{EC}^\circ_{50}/2$. It is therefore apparent that the coefficients depend solely on $p\text{EC}^\circ_{50}$.

***p*EC$_{50}$ and TCR triggering**

[8]Any scaling factor related to a choice of units (for concentration in this case) will appear as an *additive* constant after taking logarithms. Strictly speaking, transcendental functions ought to have dimensionless arguments only, lest such nuisance terms appear (certain authors insist that logarithms of dimension-bearing arguments are not valid, and according to this view, for instance, *p*H would be inadmissible). However, the empirical content of the theory only hinges on *differences* in the *p*EC$_{50}$ values, which is equivalent to taking logarithms of dimensionless quotients. This situation is similar to that of *p*H, because the physical chemistry of acids and bases only depends on differences, such as *p*H—*p*K.

We have provided a possible mechanistic underpinning for proposition (i) on the basis of TCR and pMHCI, further elucidated why data supporting proposition (i) are reported by some but not others (depending on receptor densities relative to the affinity of the TCR/pMHCI interaction). However, eqn (Trig) expresses the TCR triggering rate, whereas our experiments determine functional sensitivity in terms of an 50% response concentration. We thus need to make plausible that $p\mathrm{EC}_{50}$ is a correlate of the TCR triggering rate. We assume that a T cell exhibits a response when the intracellular signal transmitted by triggered TCRs exceeds a certain threshold:

$$S > \Theta(\text{readout}),$$

as there are generally different threshold values for different types of readout (12). Suppose that the conjunction of the T cell and the antigen-presenting cell takes place between times t_1 and t_2, and that eqn (Trig^b) applies. We then have:

$$S = \int_{t_1}^{t_2} K(t,\tau) Z_T(\tau) \lambda \exp\{-\lambda/\lambda_0\} d\tau,$$

where $K(\cdot,\cdot)$ is the signal-processing kernel of the signaling pathway. We can then allow:

$$\hat{Z}_T = \int_{t_1}^{t_2} K(t,\tau) Z_T(\tau) d\tau \text{ and } w = \lambda \exp\{-\lambda/\lambda_0\},$$

so that $S = \hat{Z}_T w$[9]. The activation condition now reads $w > \Theta(\text{r-o})/\hat{Z}_T$ or $\Theta(\text{r-o}) < \hat{Z}_T w$. A population of T cells incubated with a peptide at concentration C_P will exhibit variability in both the threshold value and the number of MHC molecules occupied by the peptide of interest. The fraction of responding cells is equal to the following probability[10]:

$$\mathbb{P}[\ln\{\Theta(\text{r-o})\} - \ln\{\hat{Z}_T\} < \ln\{w\}].$$

---

[9]This formula suggests that if *w* is smaller by (for instance) a factor two, this can be compensated by increasing the MHC density by two-fold. This is the “avidity effect” that underlies the assay in which antigen-presenting cells are prepared by incubating them with a series of concentrations of the peptide of interest. “TCR avidity” would be the obvious and natural term for *w*, but this term has no consistent usage in the literature.

[10]By the Law of Large Numbers, provided that we have a sufficient number of T cells present in each exposure, *i.e.*, the assay well.

We assume that $\ln\{\Theta(\text{r-o})\} - \ln\{\hat{Z}_T\}$ follows a normal distribution, implying that both $\ln\{\Theta(\text{r-o})\}$ and $\ln\{\hat{Z}_T\}$ are log-normally distributed, which is the appropriate standard assumption for random variables of this nature. Of particular interest is the point where the above probability equals 1/2, which happens when[11]:

$$\mathbb{E}[\ln\{\Theta(\text{r-o})\} - \ln\{\hat{Z}_T\}] = \ln\{w\}.$$

Moreover, we have $C_P = \text{EC}_{50}$ at this point, by definition. As occupancy of MHC molecules on the antigen-presenting cells depends on the incubation concentration, we again consider a Taylor expansion:

$$\mathbb{E}[\hat{Z}_T] = \sum_{\ell=0}^{\infty} \frac{k_\ell}{\ell!} C_P^\ell,$$

where we observe that $k_0 = 0$ and the first-order term will dominate, provided that C_P is sufficiently small (at higher concentrations, we expect a saturation effect, meaning that the MHC occupancy becomes less than would be expected on the basis of the first-order term alone). Accordingly, we adopt the approximation $\mathbb{E}[\hat{Z}_T] = k_1 C_P$. In addition, we have:

$$\mathbb{E}[\ln\hat{Z}_T] = \ln\{\mathbb{E}[\hat{Z}_T]\} - \frac{\mathbb{E}[\hat{Z}_T^2] - \mathbb{E}[\hat{Z}_T]^2}{2\mathbb{E}[\hat{Z}_T]^2} + \text{higher-order terms},$$

of which we retain only the first term on the assumption that the coefficient of variation of $\hat{Z}_T$ is small, which would appear to be warranted, because the number of MHC molecules on a given antigen-presenting cell is large. Combining the identities we have formulated thus far, we find:

$$\begin{aligned}
&\ln\{w\} = \mathbb{E}[\ln\{\Theta(\text{r-o})\} - \ln\{\hat{Z}_T\}] = \mathbb{E}[\ln\{\Theta(\text{r-o})\}] - \mathbb{E}[\ln\{\hat{Z}_T\}] = \\
&\mathbb{E}[\ln\{\Theta(\text{r-o})\}] - \ln\mathbb{E}[\{\hat{Z}_T\}] = \mathbb{E}[\ln\{\Theta(\text{r-o})\}] - \ln\{k_1\} - \ln\{\text{EC}_{50}\} = \\
&\mathbb{E}[\ln\{\Theta(\text{r-o})\}] - \ln\{k_1\} - (\ln 10)\log_{10}\{\text{EC}_{50}\} = \\
&\mathbb{E}[\ln\{\Theta(\text{r-o})\}] - \ln\{k_1\} + (\ln 10)p\text{EC}_{50},
\end{aligned}$$

(using $p \equiv -\log_{10}$), or:

[11]The median coincides with the mean for any symmetric distribution.

$$pEC_{50} = \frac{\ln\{k_1\}+\ln\{\lambda_0\}-\mathbb{E}[\ln\{\Theta(\text{r-o})\}]}{\ln 10} + \frac{1}{\ln 10}\ln\frac{w}{\lambda_0},$$

which is the linear relationship $pEC_{50} = \kappa + \gamma\ln\{w/\lambda_0\}$ we adopted earlier.

Choice of kinetic regime

We have now completed the chain of reasoning that connects pEC_{50} to the TCR/pMHCI dissociation rate (λ). Admittedly, this chain contains numerous steps and is somewhat tenuous at certain points. However, all that was needed for the main message of the present paper was to render proposition (i) plausible by exhibiting a relationship qualitatively similar to $pEC_{50} \propto -\ln\{\lambda/\lambda_0\}^2$. Perhaps the most critical step in the argument is the reduction of the general expression (Trig) to the special case (Trig^b). If TCR and MHC densities are such that (Trig^c) applies, the TCR triggering rate would still exhibit the optimum with respect to λ, but variation in the peptide concentration would not result in changes in the readout. The fact that we do observe this in our assay system allows us to rule out (Trig^c). However, there may be systems (T cell phenotypes) in which the TCR density is extremely low or the pMHCI density is abnormally high (e.g. genetically engineered MHCs), and (Trig^c) is the appropriate equation. There may also be systems (both TCR and MHC densities are low relative to the dissociation constant) where (Trig^a) applies, and the relationship between w and λ becomes monotone (the optimum disappears). The theory therefore accounts for studies that found this relationship to display an optimum and for studies in which it was found to be monotone. These different scenarios arise as natural special cases out of one and the same kinetics model and can in principle be verified by comparing TCR and MHC densities to the dissociation constant[12].

Readout modeling

The readout was assumed to be proportional to the fraction of responding cells in the well. This fraction is equal to the Gaussian probability:

$$\mathbb{P}[\ln\{\Theta(\text{r-o})\} - \ln\{\hat{Z}_T\} < \ln\{w\}],$$

[12]The dissociation constant here is the "two-dimensional" parameter, which differs from the "three-dimensional" value that is obtained in solution, because the molecules are confined in the z-direction orthogonal to the surface of the cell.

which is a function of incubation concentration C_P via $\mathbb{E}[\hat{Z}_T] = k_1 C_P$. Thus, the titration curve is a sigmoid with midpoint located at the $p\text{EC}_{50}$ and a midpoint slope equal to $(2\pi(\mathbb{V}[\ln\{\Theta(\text{r-o})\}] - \mathbb{V}[\ln\{\hat{Z}_T\}]))^{-1/2}$ (while these variances $\mathbb{V}[\ln\{\Theta(\text{r-o})\}]$ and $\mathbb{V}[\ln\{\hat{Z}_T\}]$ determine the shape of the sigmoid curve, they do not necessarily increase the uncertainty in the estimate of $p\text{EC}_{50}$, as discussed below). The midpoint slope can be regarded as a nuisance parameter in view of our primary aim, which is to estimate the functional sensitivity of the given TCR for the ligand at hand. Nonetheless, it is clear from the formula that this parameter contains interesting information[13]. The observations will deviate from the theoretical predicted value due to at least[14] two sources of error. One is the usual error due to apparatus, which is routinely assumed to be normal with expectation (bias) zero. The other error is binomial variance. If there are N T cells in the well and the probability of any cell responding is p, then the number of responding cells has a coefficient of variation equal to $\sqrt{p(1-p)/N}$, which shows that this source of error can be controlled by including a sufficient number of T cells in each well. If this is done, a simplified approach can be adopted, where the data are fitted on the basis of the least-squares criterion to a logistic curve (rather than the Gaussian CDF, which is numerically less convenient to work with):

$$y = y_0 + (y_{\text{max}} - y_0)/(1 + \exp\{(pC_P - p\text{EC}_{50})/\vartheta\}),$$

where y is the registered readout value, y_0 is a (non-specific) background, y_{max} is the maximum readout value, and ϑ is a midpoint slope parameter (the slope increases with decreasing ϑ). This logistic sigmoid is decreasing in pC_P, because the lowest concentrations appear the highest on the $p \equiv -\log_{10}$ scale, but we generally opt to report graphically with this scale reversed, so that the lowest concentrations are on the left and the sigmoid increases. In this simple model, we encounter three nuisance parameters (y_0, y_{max}, and ϑ) for the one parameter of interest ($p\text{EC}_{50}$), but this problem is mitigated by simultaneous least-squares estimation, where a number of curves (which may be assumed to share the

---

[13]A large value of this parameter points to low numbers of MHCI molecules on the antigen-presenting cells and/or high variability in activation thresholds for the T cells (which may itself be due to, *e.g.*, a lack of cell-cycle synchronization).

[14]Receptor kinetics limitation conditions may be different for different concentrations, which can be tackled by the more general approach given in reference (12).

values of the nuisance parameters) is used as a pooled data set during the numerical procedure used to find the least-squares minimum. For instance, practicing simultaneous estimation across a panel of 9 data sets (curves), one has 3 + 9 parameters (because each curve allows its own midpoint), which implies 12/9 = 4/3 parameters per curve (less than a two-parameter straight line).

Statistical considerations and sensitivity

The $p\mathrm{EC}_{50}$ values obtained in this manner are taken further to standard inferential statistical analysis. It may seem unusual[15] to treat a parameter estimate as a data point, but there is no fundamental methodological reason why this should not be valid. However, we may study how errors propagate. We have already commented on the components of the error on the original readout observations y. Let us assume that this error equals σ, relative to a standardized logistic curve with $y_0 = 0$, $y_{\mathrm{max}} = 1$, and midpoint zero. We focus here on the uncertainty of the midpoint parameter, because it is the parameter of primary interest and, unlike the nuisance parameters, it does not benefit from the uncertainty reduction that is obtained through the expedient of simultaneous estimation. Let us first consider simulated data sets where we have N_D data points per curve, whose pC_P values are arranged symmetrically around the midpoint:

$$pC_P \in \left\{-\Delta\frac{N_D}{2}, -\Delta\left(\frac{N_D}{2}-1\right), \ldots, -\Delta, +\Delta, \ldots, +\Delta\left(\frac{N_D}{2}-1\right), +\Delta\frac{N_D}{2}\right\},$$

where Δ is a spacing parameter (we increase the challenge by *not* having a data point at the midpoint, for reasons that will become clear shortly). We superimpose additive Gaussian noise with mean zero and standard deviation σ (in keeping with least-squares fitting, which is formally identical to maximum-likelihood estimation in the case of additive Gaussian noise). We then back-estimate the midpoint and repeat this several thousand times for each combination of parameter settings to obtain the standard deviation S_{mid} of the midpoint estimate. It is useful to compare this error to the error σ that obtains for the individual observations. Extensive numerical simulations then reveal that $S_{\mathrm{mid}}/\sigma \approx 2$ over a wide range of parameter settings, which implies that the uncertainty in the readout y translates quite stably into an uncertainty in the $p\mathrm{EC}_{50}$ estimate. We find that $S_{\mathrm{mid}}/\sigma \approx 2$ over a wide range

---

[15]This is not unusual. Laboratory instruments output “raw” data, but in reality, this is almost always itself a parameter estimate, albeit hidden from view, as it is performed by the software built into the instrument.

spanned by $\sigma \in \{0.001,1\}$, $N_D \in \{4,10\}$, and $\Delta \in \{2\vartheta, 10\vartheta\}$. For $N_D = 2$, S_{mid}/σ may be somewhat higher, rising up to $S_{\text{mid}}/\sigma \approx 2.5$. However, a dramatic increase occurs when $\Delta \approx \vartheta$ or lower. For instance, at $\Delta = \vartheta$, we find that $S_{\text{mid}}/\sigma \approx 3$ for $N_D \in \{4,10\}$ and that $S_{\text{mid}}/\sigma \approx 3.5$ for $N_D = 2$ (again these results are identical for all $\sigma \in \{0.001,1\}$), and for even lower values of the spacing parameter Δ, the ratio S_{mid}/σ quickly rises even further. These observations can be readily understood heuristically. The data set needs to cover the "bends" in the sigmoid curve, and if this condition is not fulfilled, *i.e.*, when observations are performed too close to the midpoint (as measured by the midpoint slope parameter ϑ), there is little underlying variation in the prediction, so that this "signal" is easily swamped by the noise. A sound objection against this numerical experiment is that, in practice, the data points will not be symmetrically arranged to the left and the right of the midpoint. Indeed, if this could be arranged in advance, there would be no need to perform the experiment, the purpose of which is to find the midpoint. However, the important point here is that the estimate $S_{\text{mid}}/\sigma \approx 2$ already stabilizes at $N_D = 4$, and that additional data points do not in fact lead to further improvement (as we have seen, a good choice of the spacing parameter Δ is much more critical than the number of data points, N_D). This means that as long as Δ is sufficiently large, and we have at least two data points to the left (or to the right) of the midpoint, we may trust that the quality of the estimate is not materially affected by the failure of the midpoint to lie in the precise middle of the pC_P values that were included in the experiment. If this condition is not fulfilled, the estimate is "out of range." If the EC_{50} exceeds the largest C_P included in the titration series (*i.e.*, the $p\text{EC}_{50}$ is lower than the lowest pC_P tested), an estimate can nevertheless be obtained when simultaneous estimation from "in-range" agonists furnish the estimates for the nuisance parameters. However, in this case, S_{mid}/σ is generally higher than 2, and the conditions needed to control this error become rather more stringent. Heuristically, it now becomes critical that the data points available lie sufficiently close to the midpoint to define the midpoint slope. In general, technical replicates are performed. In the standard treatment-control statistical testing scenario, one estimates the variances in the respective groups without necessarily having an insight into how these deviances are composed of observational (instrument) error as opposed to underlying true variation within the group of replicates. In the present case, the former error σ may be gleaned from the residuals of the curve fit, and the latter can then be inferred via the formula $\sigma_{X+Y} = \sqrt{\sigma_X^2 + \sigma_Y^2}$ for the standard deviation of the sum of two independent random variables X and Y.

Mathematica code to investigate error propagation (_p_EC$_{50}$ estimation)

```
siggs = {.001, .01, .1}; Nran = 3000; sp = 1;
 moll = 1/(1 + Exp[x - mid]);
ListPlot[
   Table[Table[{Np,
      StandardDeviation[
        Table[Last[
          Last[FindFit[
            Transpose[{Table[i - Np/2 - Boole[i <= Np/2], {i, Np}],
              Table[1/(1 +
                   Exp[-sp (i - Np/2 - Boole[i <= Np/2])]), {i, Np}] +
                RandomVariate[NormalDistribution[0, siggs[[s]]],
                Np]}], moll, {mid}, x]]], Nran]]/siggs[[s]]}, {Np, 2,
      10, 2}], {s, 1, Length[siggs]}], Joined -> True,
   PlotRange -> {0, 5}]
```

SUPPLEMENTARY REFERENCES

1. D. K. Cole *et al.*, Human TCR-binding affinity is governed by MHC class restriction. *J Immunol* **178**, 5727–5734 (2007).
2. D. K. Cole *et al.*, Structural mechanism underpinning cross-reactivity of a $CD8^+$ T-cell clone that recognizes a peptide derived from human telomerase reverse transcriptase. *J Biol Chem* **292**, 802–813 (2017).
3. B. Szomolay, T. Williams, L. Wooldridge, H. A. van den Berg, Co-receptor CD8-mediated modulation of T-cell receptor functional sensitivity and epitope recognition degeneracy. *Front Immunol* **4**, 329 (2013).
4. B. Malissen, An evolutionary and structural perspective on T cell antigen receptor function. *Immunol Rev* **191**, 7–27 (2003).
5. L. Wooldridge *et al.*, A single autoimmune T cell receptor recognizes more than a million different peptides. *J Biol Chem* **287**, 1168–1177 (2012).
6. H. A. van den Berg, N. J. Burroughs, D. A. Rand, Quantifying the strength of ligand antagonism in TCR triggering. *Bull Math Biol* **64**, 781–808 (2002).
7. H. A. van den Berg, D. A. Rand, Antigen presentation on MHC molecules as a diversity filter that enhances immune efficacy. *J Theor Biol* **224**, 249–267 (2003).
8. H. A. van den Berg, D. A. Rand, Foreignness as a matter of degree: the relative immunogenicity of peptide/MHC ligands. *J Theor Biol* **231**, 535–548 (2004).
9. F. den Hollander, Large deviations (Fields Institute Monographs). *American Mathematical Society* (2008).
10. A. Dembo, O. Zeitouni, Large deviations techniques and applications (2nd Edition). *Springer* (2010).
11. N. G. Van Kampen, Stochastic processes in physics and chemistry (3rd Edition). *Elsevier* (2007).
12. H. A. van den Berg *et al.*, Cellular-level versus receptor-level response threshold hierarchies in T-cell activation. *Front Immunol* **4**, 250 (2013).
